# Different ommochrome pigment mixtures enable sexually dimorphic Batesian mimicry in disjunct populations of the common palmfly butterfly, *Elymnias hypermnestra*

**DOI:** 10.1371/journal.pone.0202465

**Published:** 2018-09-12

**Authors:** Silvio Panettieri, Erisa Gjinaj, George John, David J. Lohman

**Affiliations:** 1 Department of Chemistry and Biochemistry, City College of New York, City University of New York, New York, NY, United States of America; 2 Ph.D. Program in Chemistry, Graduate Center, City University of New York, New York, NY, United States of America; 3 Biology Department, City College of New York, City University of New York, New York, NY, United States of America; 4 Ph.D. Program in Biology, Graduate Center, City University of New York, New York, NY, United States of America; 5 Entomology Section, National Museum of the Philippines, Manila, Philippines; USDA Agricultural Research Service, UNITED STATES

## Abstract

With varied, brightly patterned wings, butterflies have been the focus of much work on the evolution and development of phenotypic novelty. However, the chemical structures of wing pigments from few butterfly species have been identified. We characterized the orange wing pigments of female *Elymnias hypermnestra* butterflies (Lepidoptera: Nymphalidae: Satyrinae) from two Southeast Asian populations. This species is a sexually dimorphic Batesian mimic of several model species. Females are polymorphic: in some populations, females are dark, resemble conspecific males, and mimic *Euploea* spp. In other populations, females differ from males and mimic orange *Danaus* spp. Using LC-MS/MS, we identified nine ommochrome pigments: six from a population in Chiang Mai, Thailand, and five compounds from a population in Bali, Indonesia. Two ommochromes were found in both populations, and only two of the nine compounds have been previously reported. The sexually dimorphic Thai and Balinese populations are separated spatially by monomorphic populations in peninsular Malaysia, Singapore, and Sumatra, suggesting independent evolution of mimetic female wing pigments in these disjunct populations. These results indicate that other butterfly wing pigments remain to be discovered.

## Introduction

Mimicry, in which one species imitates another in appearance, odor, behavior, or some other trait, is taxonomically widespread and takes diverse forms [1]. Perhaps the simplest categorization discriminates Batesian mimicry, in which a mimetic species resembles a model species that is avoided by predators [2], and Müllerian mimicry, in which two or more species that are avoided by predators converge on a similar phenotype [3]. In butterflies, model species are often avoided by predators because host plant-derived compounds render them unpalatable [4], but model species might be avoided for other reasons, such as insuperably evasive flight maneuvers [5, 6]. Within a given area, predators learn to associate unpleasant taste or uncatchable species with particular visual phenotypes. Batesian mimics enjoy reduced predation by being mistaken for another species that predators learn to avoid, while co-occurring Müllerian mimics benefit from sharing the cost of educating predators on the association between the shared phenotype and its undesirability [1]. Mimicry “rings” occur when three or more species converge on a similar pattern, and may include both Batesian and Müllerian mimics [7].

Co-occurring Müllerian mimics are regarded as mutualists, whereas Batesian mimics are thought to be parasites of their models [8]. Although both forms of mimicry generate phenotypic diversity within and between species, differing selection pressures are likely to lead to this novelty via different mechanisms [9, 10]. Müllerian mimetic species are rarely if ever sexually dimorphic, but a single butterfly species can be polymorphic, with markedly different wing patterns shared by both sexes in different locales where they join different mimicry rings [8]. Some co-distributed and wide-ranging Müllerian mimics, such as *Heliconius cydno* and *H*. *melpomene*, have the same wing patterns where they co-occur, but the shared patterns differ markedly in different locales [11]. In addition to monomorphic species, some Batesian mimics have sex-limited mimicry in which a single species has dissimilar female forms mimicking different models, while males are not mimetic and are uniform in appearance throughout their range [12].

Mimetic and other types of visual signals in butterflies are made possible by structural color and pigments of wing scales. Structural color results when nanoscale ridges and other physical aspects of scales selectively reflect and refract different wavelengths of light [13]. Most butterfly wing colors are due to pigments, and the majority of butterfly wing pigments are either melanins, flavonoids, pterins, and ommochromes [14–17], though other compounds may impart color in some taxa [18, 19]. Ommochromes are synthesized in a metabolic pathway that transforms tryptophan into red, orange, or yellow pigments with the aid of several enzymes and transporter molecules [20, 21]. Ommochromes are ubiquitous filtering pigments in insect eyes but also add color to silkworm eggs and the bodies of spiders, dragonflies, and squid [17, 22–24]. Within butterflies, the deployment of ommochromes as wing pigments is known only from the Nymphalidae [14, 20, 25].

The genetic, genomic, and developmental basis of butterfly wing patterns are the subject of much contemporary research. This work often focuses on identifying genomic regions that produce wing pigments/patterns or spatial patterns of gene expression in developing wings that result in the mosaic of colors in a butterfly’s wing pattern [26–32]. However, the chemical identity of wing pigments has received less attention. Although there are *ca*. 18,500 butterfly species [33], wing pigments from a limited number of butterfly species have been characterized chemically [13, 14, 16, 34, 35]. Most studies that investigate ommochrome pigments in butterflies focus on a small number of species with pigments that were characterized many years ago [19, 35–38]. Few studies seek to explore the full extent of butterfly diversity to look for novel pigments.

**Fig 1:**
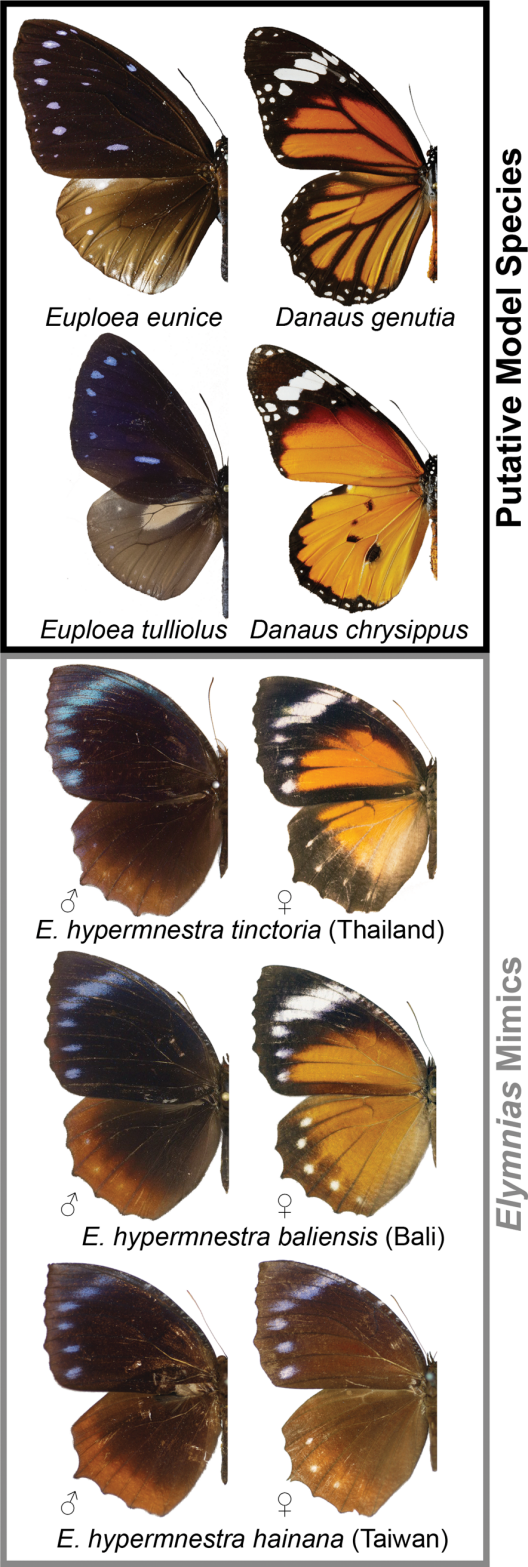
*Elymnias hypermnestra* mimics and their model species. Sexually dimorphic male and female specimens of *E*. *hypermnestra tinctoria* from Chiang Mai, Thailand, and *E*. *h*. *baliensis* from Bali, Indonesia, compared with a monomorphic subspecies, *E*. *h*. *hainana* from Taiwan, and putative model species. Used under a CC BY license with permission from Shen-Horn Yen, the Taiwan Forestry Research Institute, and the Museum of Comparative Zoology.

**Fig 2:**
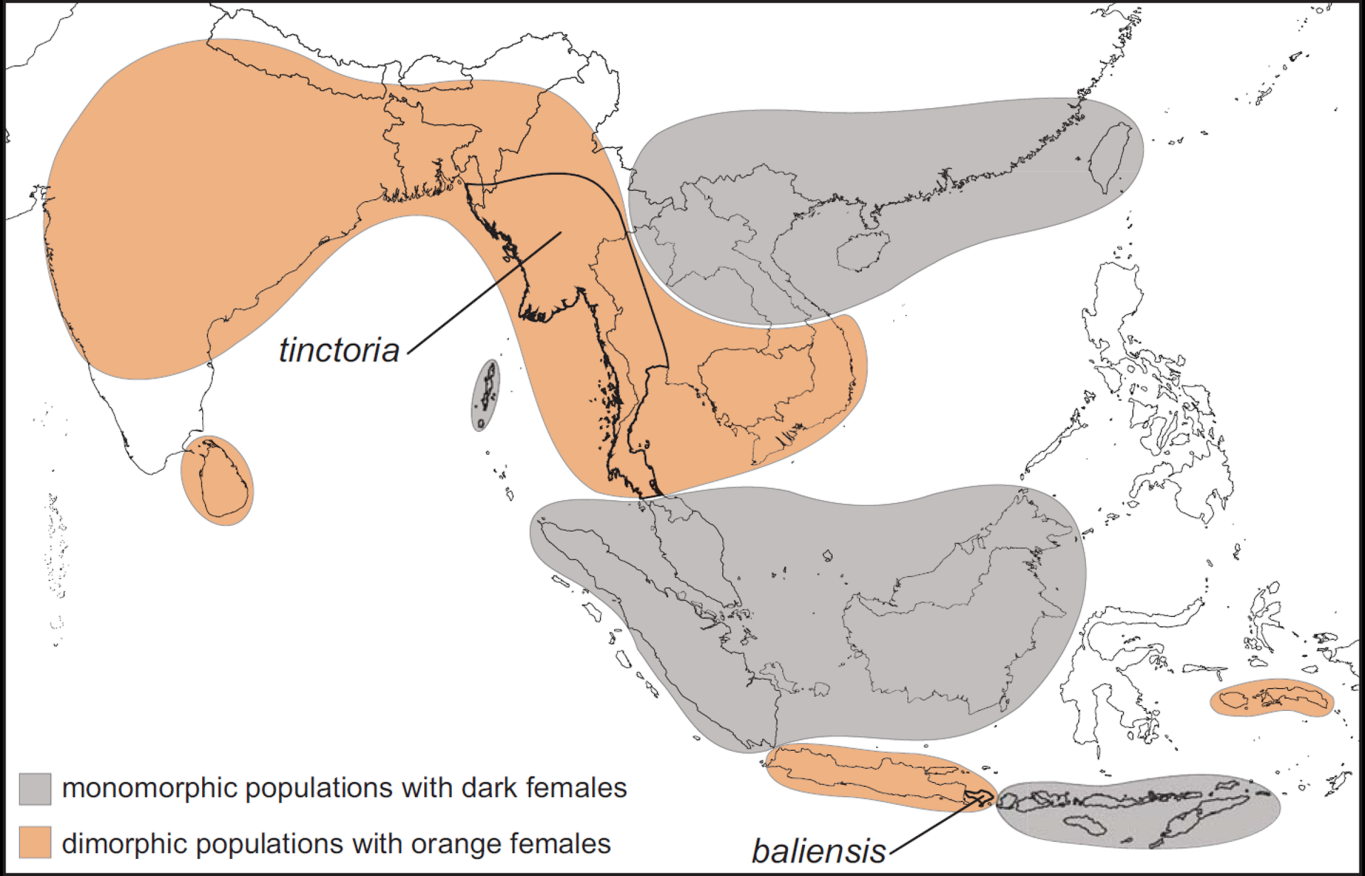
Geographical distribution of sexually dimorphic and monomorphic *Elymnias hypermnestra* populations. The geographic ranges of the two subspecies sampled in this study, *E*. *h*. *tinctoria* and *E*. *h*. *baliensis*, are indicated.

We investigated the chemical identity of orange ommochrome wing pigments in female *Elymnias hypermnestra* butterflies (Lepidoptera: Nymphalidae: Satyrinae), a Batesian mimetic species in which some populations are sexually dimorphic (Fig 1). This is the most widely distributed species in the genus *Elymnias*, and its 23 subspecies range throughout most of South and Southeast Asia (Fig 2) [39]. The larvae feed on a variety of palm species (Arecaceae) [40], and adults of *El*. *hypermnestra hainana* are readily consumed by naïve avian predators in captivity (S.-H. Yen, *unpublished results*), indicating that the species does not seem to acquire or produce noxious defensive chemicals that might render them unpalatable to predators. However, males and females of the species are protected by resemblance to different unpalatable model species that visually-orienting predators learn to avoid [41–43]. The wings of every male *El*. *hypermnestra* subspecies have a dark brown/black ground color with a bluish apical band on the forewing; some populations also have light brown tornal bands on the hindwings. This phenotype resembles several species of *Euploea* butterfly (Nymphalidae: Danainae) including *Eu*. *tulliolus*, *Eu*. *sylvester*, and probably *Eu*. *eunice* (Fig 1) [42]. The differences between models and mimics might seem obvious to a human observer (Fig 1), but the predators acting as selective agents in nature may have visual discriminatory abilities that differ from humans [44–46]. In addition, the mimetic ruse is often aided by motion blur caused by beating wings and behavioral mimicry in which the mimetic species imitates the height, speed, and other characteristics of the model’s flight [6, 47, 48].

Wing patterns of female *Elymnias hypermnestra* are variable. In eastern Indochina, peninsular Malaysia, Borneo, Sumatra, and the Lesser Sundas east of Wallace’s Line, females resemble males, and populations are more or less monomorphic. In South Asia, western Indochina, Java, Bali, Seram, and Buru, females have orange wings rimmed with black and a white subapical band on the forewings, which closely resembles wing patterns of the unpalatable species *Danaus chrysippus* and *D*. *genutia* (Nymphalidae: Danainae) (Figs 1 and 2) [42]. The geographic distribution of *El*. *hypermnestra* is within the range of multiple *Euploea* and *Danaus* putative model species [39, 49], which is presumed necessary for the persistence of Batesian mimetic species [50–52]. Noxious defensive chemicals are sequestered by larvae of these *Euploea* and *Danaus* species when they feed on their Apocynaceae and Moraceae host plants [53, 54], and different compounds are sequestered by species in these two genera [4, 55]. Adult males of some species are further defended by imbibing pyrrolizidine alkaloids [56]. These compounds provide protection from predators throughout the larval, pupal, and adult stages, and the distinctive color patterns that advertise their unpalatability are copied by a variety of Müllerian and Batesian mimics in several butterfly and moth families, including other *Elymnias* species [42, 50, 57].

Since the shade of orange on wings of specimens from Thai and Balinese populations differ, we hypothesized that different pigments are responsible for wing coloration in the two populations. Differences could suggest local adaptation to different models and—in concert with other data—parallel evolution of orange color implicating Batesian mimicry as a selective force generating biochemical novelty.

## Materials and methods

### Specimen acquisition and pigment extraction

Adult female *Elymnias hypermnestra baliensis* specimens were obtained from a wild population in Bali, Indonesia (8.5°S 115.3°E), and adult female *Elymnias hypermnestra tinctoria* specimens were obtained from a wild population in Chiang Mai, Thailand (18.9°N 99.0°E). *Elymnias hypermnestra* is neither endangered nor protected in Indonesia or Thailand. Specimens were not collected in protected areas, and field collection and export permits, if required, were obtained from the relevant authorities. Specimen collection in Indonesia was pursuant with permits from Kementerian Riset Dan Teknologi, Lembaga Ilmu Pengetahuan Indonesia, Markas Besar Kepolisian Negara Republik Indonesia, Kementerian Dalam Negeri Republik Indonesia, and other relevant Indonesian authorities. Export of specimens from Indonesia was conducted under the terms of a Memorandum of Understanding and a Material Transfer Agreement between the City College of New York and Lembaga Ilmu Pengetahuan Indonesia. Collection of non-protected, non-CITES species outside of protected areas in Thailand did not require permits, and export of such species from Thailand also does not require permits. However, DJL's work in Thailand was conducted with permits from สภาวิจัยแห่งชาติ and กรมอุทยานแห่งชาติ สัตว์ป่า และพันธุ์พืช. In addition, optional documentation was obtained from สำนักงาน CITES of กรมอุทยานแห่งชาติ สัตว์ป่า และพันธุ์พืช confirming that exported specimens are not CITES-listed.

Due to the instability of ommochrome pigments in light as illustrated by Bolognese *et al*. [58], we were careful during pigment extraction to minimize the possibility of (photo-) oxidation and ensure preservation of the naturally occurring pigments. We deoxygenated all solvents prior to extraction by bubbling pure nitrogen in the stock bottle for 30 min. Every step in our extraction protocol was performed either in the dark (by wrapping aluminum foil around vessels containing extracts) or, when necessary, in a fume hood with dimmed lights. We kept the samples at 4°C during the extraction procedure and stored them at -20°C prior to analysis.

Two extracts were made: one from pooled wings of 10 female *E*. *h*. *baliensis* specimens (4 wings per butterfly, 40 wings per extraction), and another from 4 female *E*. *h*. *tinctoria* specimens (we were unable to obtain 10 females of this subspecies). The orange regions of all four wings (both forewings and both hindwings; Fig 1) were excised with scissors and macerated in a fume hood with dimmed lights (to minimize photo-oxidation) in a single 1.5 ml tube with 500 μL of deoxygenated 70% methanol in water before being ground with a plastic pestle, following the protocol of Nijhout [37]. Each sample mixture was then sonicated at room temperature for 15 minutes using a Branson 5510 ultrasonicator (bransonic.com) in the dark; the tube was wrapped in aluminum foil, vortexed at maximum speed for 2 min, and centrifuged for 5 min at 8000 rpm. The supernatant was discarded and the tissue was dried under a gentle flow of nitrogen in the dark at 4°C. The wing tissue was then placed in a solution of deoxygenated 0.1% HCl_conc_ in methanol (v/v), and ground using a pestle at 4°C in a fume hood with dimmed lights to prevent esterification reactions of the target compounds in solution [59]. The sample was wrapped in aluminum foil, sonicated at room temperature for 5 min, vortexed, and centrifuged as before. The supernatant was then recovered and filtered on a PES microfilter (EMD Millipore, pore size 0.22 μm) in a fume hood with dimmed lights. The extract, which was dark brown, was stored at -20°C until analysis. An initial, unsuccessful attempt to extract ommochromes strictly followed extraction methods used by Nijhout [37] and did not include ultrasonication and vortexing; we found these steps were necessary for successful pigment extraction. Pooling multiple individuals into a single sample prevented assessment of individual variation, but was necessary to obtain sufficient quantities of these unknown pigment compounds for analysis.

### HPLC-MS analyses

Samples were analyzed on an Agilent 1290 Infinity high performance liquid chromotography system coupled with an Agilent 6550 Q-ToF (Time-of-Flight) mass spectrometer (MS) and a UV-Vis detector, which was set to 450 nm. The mass spectrometer used an Agilent Poroshell 120 SB-C18 column (2.7 μm, 2.1 x 50 mm) at 40°C and a linear gradient of 5–95% methanol in water (0.1% formic acid) with a flow rate of 0.4 mL/min. MS analyses were run with the following parameters: VCap = 3500 V, Gas temp = 250°C. MS/MS analyses were run with CE = 30 V.

## Results

We identified six ommochrome pigments in *Elymnias hypermnestra tinctoria* and five in *E*. *h*. *baliensis*; only two compounds were found in the wings of both subspecies. With the exceptions of xanthommatin and decarboxylated xanthommatin [17, 22, 23, 60], which were found in *E*. *h*. *tinctoria* and *E*. *h*. *baliensis*, respectively, none of the compounds have apparently been identified before.

**Fig 3:**
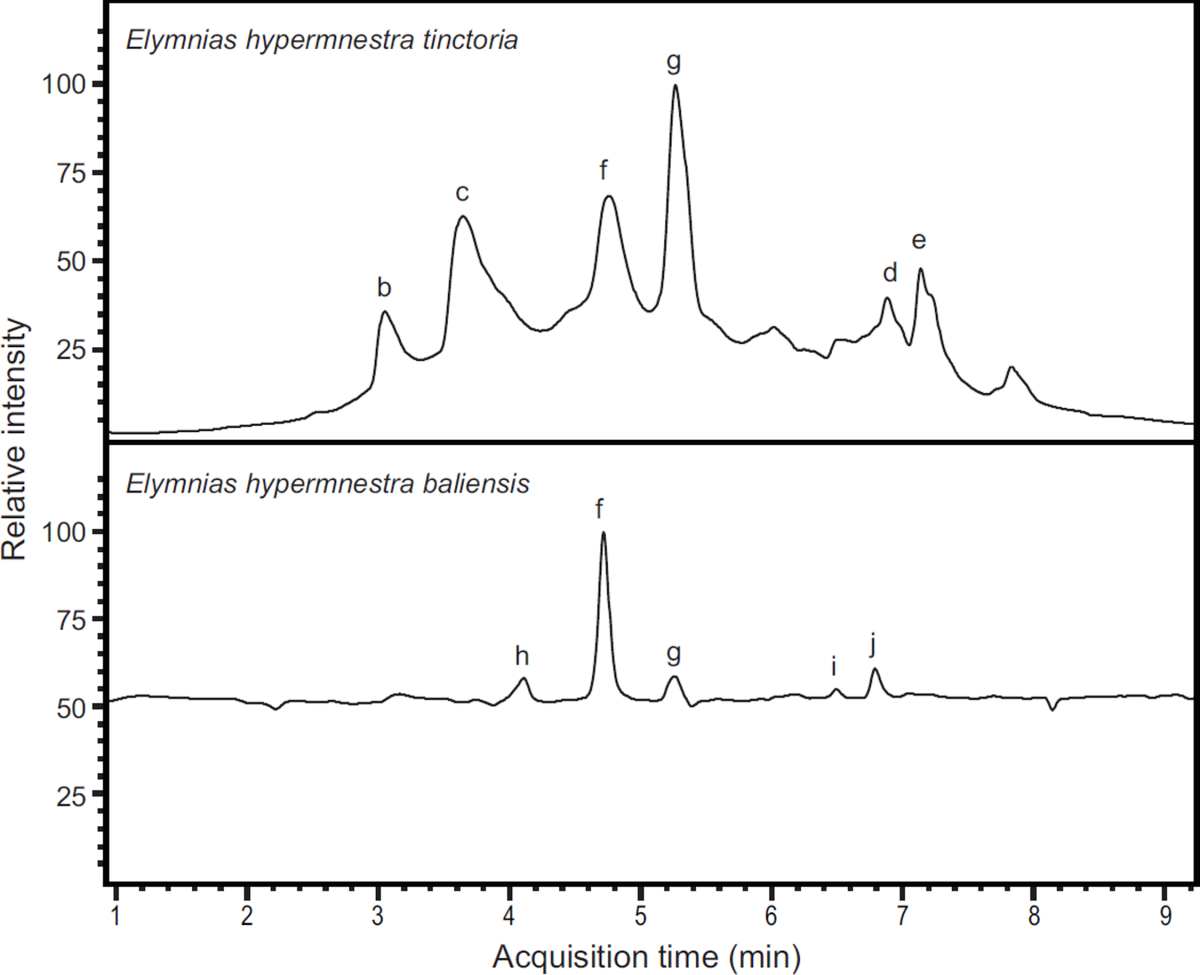
Chromatograms of pigment mixtures extracted from orange wings of female *Elymnias hypermnestra tinctoria* (upper), *and E*. *h*. *baliensis* (lower). Letters (b-j) refer to ommochrome compounds identified from each extract (Fig 4).

**Fig 4:**
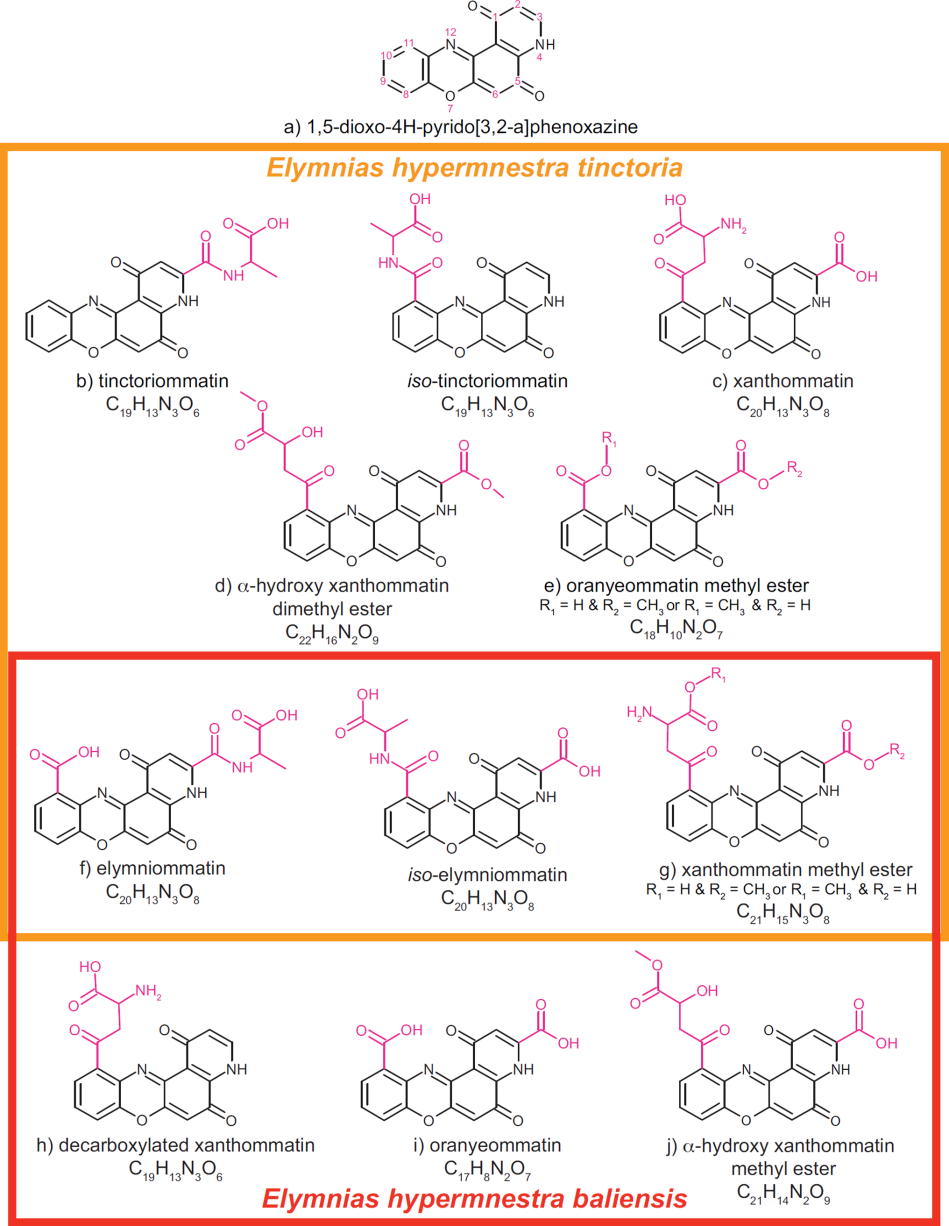
Hypothesized chemical structures and trivial names of ommochrome pigment compounds isolated from the wings of orange female *Elymnias hypermnestra*. All compounds share a common 4-ring core (a); modifications to this core are indicated in pink. Six compounds were isolated from *E*. *h*. *tinctoria* in Thailand (b-g) and five were isolated from *E*. *h*. *baliensis* from Bali, Indonesia (f-j). Two compounds (f-g) were found in both populations. With the exception of xanthommatin (c) and decarboxylated xanthommatin (h) [17, 22–24, 60], all compounds are characterized for the first time in this study. The precise isomeric structure of tinctoriommatin (b) and elymniommatin (f) could not be determined with certainty; mass spectra and alternative structures of the isomers of are reported in S1 File.

The differences in composition were apparent in the LC-MS chemical profiles of wing pigments from the two subspecies (Fig 3). We expected to find pigment molecules that shared the tetracyclic heteroaromatic ommochrome core 1,5-dioxo-4H-pyrido[3,2-a]phenoxazine (Fig 4A), which is biosynthesized using tryptophan as a precursor [20, 21]. Our interpretation of each compound’s mass spectrum reasonably assumes that its structure includes this core. To elucidate the structures with high confidence, we took advantage of HRMS to determine empirical formulae and predict putative structures, and used MS/MS to gain more detailed structural information.

We isolated and characterized six different pigments in *E*. *h*. *tinctoria* (Figs 3 and 4), including xanthommatin (Fig 4C). The mass spectrum of peak b (Fig 3) has a molecular ion ([M+H]^+^) of 380.09 Da that corresponds to the molecular formula C_19_H_13_N_3_O_6_. The MS/MS spectrum yields evidence for the presence of an alanine residue (fragment 307.07 m/z; Figure b in S1 File) that—combined with the inferred molecular formula—suggests structures lacking the carboxylic acid moiety in either position 11 or position 3 (Fig 4B). We cannot determine with certainty which of these compounds (or both) are present in our sample. We refer to these newly discovered pigments as tinctoriommatin and *iso*-tinctoriommatin, after the subspecific name of the butterfly population from which the compounds were isolated.

The mass and the corresponding empirical formula for peak c (Fig 3) precisely correspond with xanthommatin. With a retention time of 3.6 min, the corresponding mass spectrum has the fragments 317.1 m/z (loss of the side chain in position 11 plus neutral H_2_O loss) and 307.1 m/z (loss of the side chain in position 11 only; Figure c in S1 File). These data are consistent with the structure of xanthommatin (Fig 4C). Interestingly, the mass relative to peak f (retention time 4.7 min; Fig 3) is the same as xanthommatin. MS/MS analysis revealed that it corresponds with high confidence to an isomeric form having of an alanine residue attached to the carboxylic acid in position 3 via an amide bond (Fig 4F). Also, the keto-amino acid portion in position 11 seems to have undergone oxidative cleavage, giving rise to a carboxylic acid substituent. The fragments 305.05 and 317.05 in the MS/MS spectra (Figure f in S1 File) are clearly the result of the combined loss of the alanine portion via the labile amide cleavage in two different positions and either CO_2_ or H_2_O. Since this compound was found in both populations of *Elymnias hypermnestra* that we examined, we named it elymniommatin after the genus name. Owing to the limits of MS/MS-based structural characterization, we cannot discriminate between this compound and an isomer of xanthommatin that differs from this structure only in the position of the alanine residue. We term that former compound elymniommatin and the latter *iso*-elymniommatin, which carries the amide bond with the carboxylic acid moiety in position 11 and a free carboxylic acid in position 3.

The mass of peak g (C_21_H_15_N_3_O_8_; Fig 3) differs from xanthommatin by only 14 Da (+ CH_2_). The only structural modifications that would be biosynthetically reasonable involve methylation of either one of the carboxylic acids present in xanthommatin (Fig 4G,) giving rise to a methyl ester moiety. The fragment [M-MeOH]^+^ with m/z = 407.05 supports this proposed structure (Figure g in S1 File). We named this compound xanthommatin methyl ester, which was isolated from both populations.

Peak d (Fig 3) has a mass of 453.09 Da with the corresponding empirical formula C_22_H_16_N_2_O_9._ A thorough analysis of the fragments obtained via MS/MS supports the hypothesis that the molecule is the doubly methylated version of xanthommatin (Fig 4D) with the substitution of the amino group in alpha position with a hydroxyl radical, leading us to name this compound α-hydroxy xanthommatin dimethyl ester. We observed neutral H_2_O loss from the hydroxyl group in alpha position (435.1 m/z) in its MS/MS spectrum, the loss of the fragment -COOCH_3_ (375.1 m/z), and the mass 304.0 m/z arising from the loss of the latter fragment along with part of the side chain (Figure d in S1 File).

The MS/MS spectrum of peak e (Fig 3) shows a neutral H_2_O loss fragment (349.04 m/z) along with another intense fragment with mass 289.02 m/z that can be associated to the loss of CO_2_+MeOH (M-76.1 m/z, Figure e in S1 File). By combining these findings with its empirical formula C_18_H_10_N_2_O_7_, we propose pigments containing either a methyl carboxylate in position 11 and a free carboxylic acid in position 3 or a free carboxylic acid in position 11 and a methyl carboxylate in position 3 (Fig 4E). We call this compound oranyeommatin methyl ester, as it is the methyl ester of the compound in peak i, which we term oranyeommatin.

The orange-brown pigment fraction of *E*. *h*. *baliensis* contained five distinct compounds (Fig 3) with masses and empirical formulae that were consistent with xanthommatin derivatives. Interestingly, we did not detect xanthommatin itself, but variously substituted xanthommatin-derived molecules: elymniommatin/*iso*-elymniommatin and xanthommatin methyl ester were also found in *E*. *h*. *tinctoria* wing extracts (Fig 4F and 4G).

With a formula of C_19_H_13_N_3_O_6_ and [M+H]^+^ = 380.09, peak h (Fig 3) appears to be an analog having the keto-amino acid portion in position 11 and lacking the carboxylic group in position 3 (Fig 4H). Accordingly, the fragment with mass 291.04 m/z corresponds to the loss of the aminoacidic portion, whereas the fragment 363.06 and 334.08 m/z arise from H_2_O and CO_2_ loss, respectively (Figure h in S1 File). This pigment, decarboxylated xanthommatin, has previously been isolated from several arthropods and the chromatophores of a squid [17, 22, 23, 60].

Peak i (Fig 3) is the lightest of the detected pigments with [M+H]^+^ = 353.04 m/z and the formula C_17_H_8_N_2_O_7_. Based on this information, we proposed that the compound’s substituents are merely two carboxylic acid groups in position 3 and 11 (Fig 4I). The MS/MS spectrum provides little structural information as expected due to the high stability of the chromophore core but it presents two significant fragments, namely 307.03 m/z and 289.02 m/z, which correspond to [M-CO_2_]^+^ and [M-CO_2_-H_2_O]^+^ (Figure i in S1 File). These data support the presence of two carboxylic acid groups and exclude the possibility of more complex substituents on the central core. We named this pigment oranyeommatin after the Balinese and Bahasa Indonesia word for orange: oranye.

The structure of the compound in peak j (Fig 3), which has the formula C_21_H_14_N_2_O_9,_ is related to xanthommatin methyl ester. Compared to xanthommatin methyl ester, the empirical formula for this molecule differs in having one additional oxygen and one fewer nitrogen atom (Fig 4J). Assuming that the chromophore core is not affected, we hypothesize that the amino acidic portion underwent a deamination reaction that introduced a hydroxyl group in the alpha position along the side chain in position 11. Accordingly, a fragment with m/z = 307.07 arises from the cleavage of the aromatic carbon-carbonyl carbon bond and the loss of the whole side chain containing the methyl carboxylate moiety (Figure j in S1 File). Unlike some of the previously described molecules, this structure can be identified unambiguously as having the free carboxylic acid moiety in position 3. We name this novel pigment α-hydroxy xanthommatin methyl ester.

NMR analysis was considered to further characterize each pigment and determine their structure with greater certainty, even though determination of positional isomers would not be possible with this technique. Unfortunately, the logistical difficulty of collecting a sufficiently large number of females from both butterfly populations in the field prevented us from being able to successfully perform such analysis. We believe, however, that the semi-definitive nature of some of the structures reported here does not diminish the relevance of our findings.

## Discussion

Mimicry begets wing pattern diversity in many butterfly taxa [10], but the phenotypic diversity found in the genus *Elymnias* is extraordinary in several respects. One peculiarity of the genus is the prevalence of sexually dimorphic mimicry, in which both males and females mimic different models [42]. This is rare outside of *Elymnias*, though there are a few examples [61, 62]. Another characteristic of the group is apparently convergent or parallel evolution of strikingly similar wing patterns in allopatric or disjunct non-sister species to facilitate mimicking the same model in different locales, viz. mimicry of female *Euploea mulciber* by female *Elymnias casiphone*, *El*. *casiphonides*, *El*. *saueri*, and others [42]. In addition, with 53 recognized species, all but a handful of which are mimetic, *Elymnias* is one of the largest clades comprised primarily of Batesian mimics (similar clades include *Hypolimnas* and some *Papilio* lineages) [62, 63].

The genetic and developmental relationship between sexual dimorphism and female-limited mimicry in Batesian mimics is not firmly established [64], and sexual dimorphism in different Old World Batesian mimetic *Papilio* species have evolved independently via different mechanisms [29, 32]. This suggests that mimetic adaptations of Batesian mimetic butterflies might stem from a variety of different mechanisms, and the phenotypic diversity and species richness of *Elymnias* makes it well suited to studying the ecology and evolution of Batesian mimicry including phenotypic convergence and divergence.

The presence of different ommochrome mixtures pigmenting the orange wings of *Elymnias hypermnestra* females in geographically disjunct locales may result from different environmental influences and/or independent evolution on different landmasses. For example, different host plant species or other factors during larval development may trigger different developmental pathways resulting in biosynthesis of different pigment molecules. Alternatively, different selective regimes in the tropical (Bali) and subtropical (Thailand) habitats where these organisms are found may be involved. For example, different populations of model *Danaus* species may differ in color, and female *E*. *hypermnestra* mimics may be adapting to local variation in their model species.

Preliminary molecular phylogenetic analysis of this species indicates that each of these subspecies with orange females is more closely related to monomorphic subspecies with dark females, and that the species as a whole is sister to *Elymnias caudata* in southern India, which also has orange, *Danaus*-mimicking females [42]. It is thus likely that orange females are ancestral in *E*. *hypermnestra*, and our results suggest that the ability to synthesize orange pigments in females was either lost multiple times or lost and regained in different lineages. Perhaps the ommochrome pathway is silenced in lineages with dark females through one or more mutations, and back mutation(s) have restored pigment synthesis in populations with orange females.

The presence of unique compounds in each population suggests parallel evolution: evolutionary elaboration of a core developmental pathway for producing ommochrome pigments may have occurred in one or both of these populations [65]. A recent review of color in nature concluded that convergent color phenotypes commonly arise in parallel [66], and this seems a plausible explanation for the inter-population differences that we find. An alternative explanation for the presence of both orange and dark females in the species posits that orange pigmentation is still produced in dark females but is masked by melanin-derived pigments. We see little phenotypic evidence for this.

It is also possible that differences between populations may not be adaptive. Perhaps only the two compounds shared between the populations we examined, elymniommatin/*iso*-elymniommatin and xanthommatin methyl ester, are all that is needed for female wings to appear orange, and the other compounds detected are not relevant to an adaptive phenotype. However, xanthommatin has been chemically characterized previously and is known to pigment wing scales orange, suggesting that non-shared compounds such as xanthommatin are also part of the adaptive phenotype.

Our results suggest that visually similar mimetic phenotypes have a different but related biochemical basis, likely resulting from decoupled evolutionary processes in disjunct populations. The large number of novel compounds that we describe suggests that there may be much pigment diversity waiting to be discovered in other mimetic butterflies. Co-occurring Batesian mimics are often members of different families or subfamilies, whereas Müllerian mimetic species are often closely related to each other—members of the same genus or tribe. Because Müllerian co-mimics are more closely related to each other, color and pattern similarity is more likely to result from a common developmental basis inherited from a recent common ancestor or from horizontal transfer. Adaptive hybridization between co-occurring Müllerian mimetic *Heliconius* species has resulted in phenotypic similarity from horizontal transfer of wing-patterning genes, rather than convergent evolution [67, 68]. However, because of the evolutionary distances between Batesian mimics and their models, hybridization is unlikely to result in viable offspring; convergent evolution of similar phenotypes is thus more likely.

**Fig 5:**
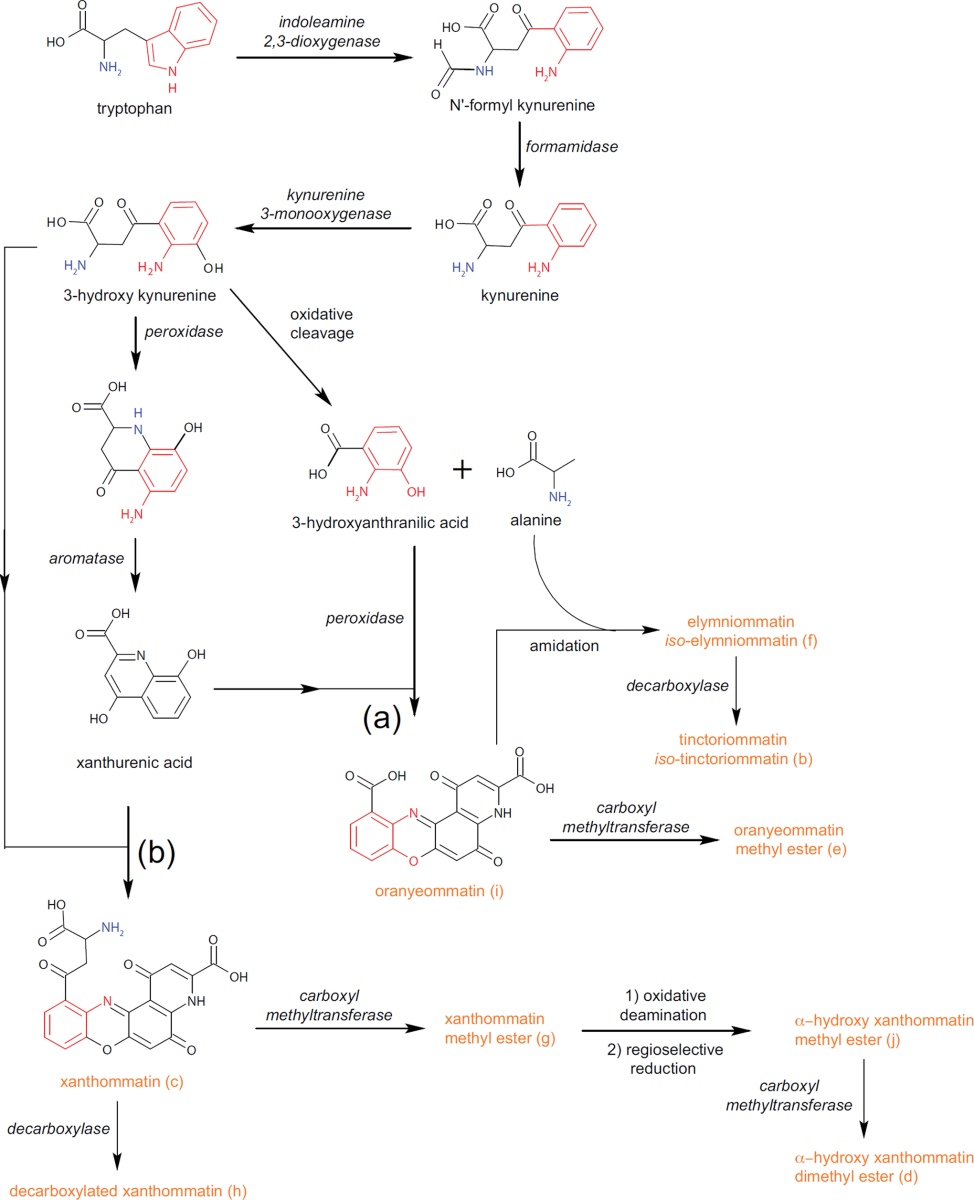
Hypothesized biochemical pathways for synthesis of the ommochrome compounds identified in this study.

We propose two main biosynthetic routes for the novel pigments found in this study. In the first (Fig 5A), the putative molecular scaffold is represented by oranyeommatin that bears two carboxylic acid moieties as side chains. This pigment is likely derived from an oxidative condensation between 3-hydroxyanthranilic acid and xanthurenic acid. While oranyeommatin methyl ester is the result of a monomethylation reaction, elymniommatin, tinctoriommatin, and their isomeric analogs contain an alanine residue linked to the chromophoric core via an amide bond. Interestingly, along with 3-hydroxyanthranilic acid, alanine results from the oxidative cleavage of 3-hydroxykynurenine (Fig 5). We speculate that during the progression of wing pigmentation, this amino acid is first produced via 3-hydroxykynurenine’s degradation and is then reincorporated into more complex molecules, therefore contributing to the wide array of pigments that characterize *Elymnias hypermnestra*.

In the second biosynthetic route (Fig 5B), both the keto-aminoacidic side chain of xanthommatin and its carboxylic acid undergo a series of modifications. The loss of the aromatic carboxylic acid gives rise to decarboxylated xanthommatin. Methyl esterification of the aliphatic carboxylic acid leads to xanthommatin methyl ester. Upon its oxidative deamination that generates a putative alfa-keto derivative and its successive regioselective reduction to hydroxyl group, α-hydroxy xanthommatin methyl ester is formed. Finally, a second methylation of the remaining carboxylic acid group yields α-hydroxy xanthommatin dimethyl ester.

The suite of ommochrome compounds found in each butterfly population does not perfectly correspond to either of the two hypothesized biosynthetic pathways (Fig 5). For example, we hypothesize that oranyeommatin methyl ester is produced through the modification of oranyeommatin, but the latter compound was not found in the wings of *E*. *h*. *tinctoria*, from which oranyeommatin methyl ester was isolated. This might be because trace amounts of oranyeommatin were present in *E*. *h*. *tinctoria*, but could not be distinguished analytically from background noise. Alternatively, these ommochrome compounds might be produced by more elaborate pathways than we posit.

We cannot completely exclude the possibility that the mixtures of pigment compounds we identified are breakdown products resulting from storage or processing of the samples. However, we regard this as unlikely. Regardless of their age, pinned museum specimens are consistently different in color between the two subspecies, suggesting true, stable differences in the underlying pigment molecules. In addition, it would seem reasonable that if degradation of the same parent ommochrome molecule(s) occurred over time in samples from the two populations, the resulting mixtures of breakdown products would be similar to each other, which is not what we found. Indeed, several compounds unique to a single population are larger and more complex than the components shared by both populations—these are unlikely to result from degradation of a parent molecule. The relative stability of ommochrome molecules and our care in processing and storing the samples to minimize photo-oxidation or non-specific chemical modifications bolster confidence that the compositional differences we observe between populations are not artifactual.

This novel comparative study suggests directions and taxa for further research on novel pigment compounds: Are orange pigments present in the wings of males and/or females in “monomorphic” populations but covered by melanin pigments? Do the putative models *Danaus genutia* and *D*. *chrysippus* produce the same orange pigments as co-occurring *E*. *hypermnestra* females? *Elymnias hypermnestra* and its sister taxon *E*. *caudata* are the only members of their genus with orange pigmentation, but the evolutionary origin of orange wing pigments predates the origin of this group [69]. What genetic mechanisms may be involved in silencing this pathway in an ancestor of *Elymnias* and its reactivation in this clade? Perhaps most importantly, a strongly supported intraspecific phylogeny of this group will establish whether dimorphic populations from the two disjunct regions with orange females—mainland Asia and Java/Bali—are more closely related to each other than to monomorphic populations that are geographically adjacent.

The discovery of xanthurenic acid in *Junonia coenia* butterfly wings prompted Daniels and Reed [34] to conclude that the ommochrome synthesis pathway in butterflies must diverge from the model developed for *Drosophila* eyes by Linzen [20], which could not accommodate synthesis of that molecule. Similarly, our findings prompt novel hypotheses for the biosynthetic pathways capable of generating these molecules.

In sum, the presence of different ommochrome compounds in disjunct populations suggests that parallel evolution in mimetic butterflies can result in novel wing pigments. With over four dozen highly varied Batesian mimetic species distributed across the world’s largest archipelago [70], the genus *Elymnias* might prove to be a wellspring of novel butterfly wing pigments.

## Supporting information

S1 File Mass spectra of ommochrome compounds identified in this study. Letters associated with each trivial name refer to the same compound in Figs 3 and 4; (a) is therefore omitted from this figure.
(PDF)
