# Supplementary material for: Different ommochrome pigment mixtures enable sexually dimorphic Batesian mimicry in disjunct populations of the common palmfly butterfly, Elymnias hypermnestra
Source: PLoS One. 2018 Sep 12;13(9):e0202465. doi: 10.1371/journal.pone.0202465 (PMC6135364; doi:10.1371/journal.pone.0202465)
Supplement: S1 File — Letters associated with each trivial name refer to the same compound in Figs 3 and 4; (a) is therefore omitted from this figure. (PDF) [file pone.0202465.s001.pdf]

**S1 Fig. Mass spectra of ommochrome compounds identified in this study.** Letters associated with each trivial name refer to the same compound in Figures 3 and 4; (a) is therefore omitted from this figure.

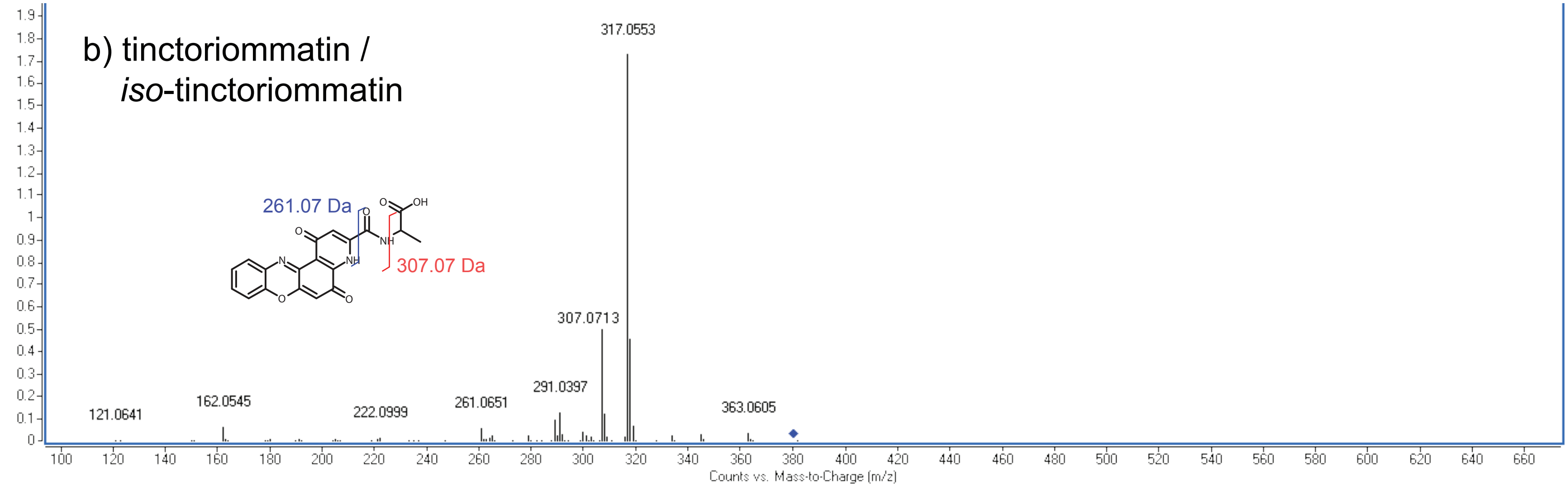


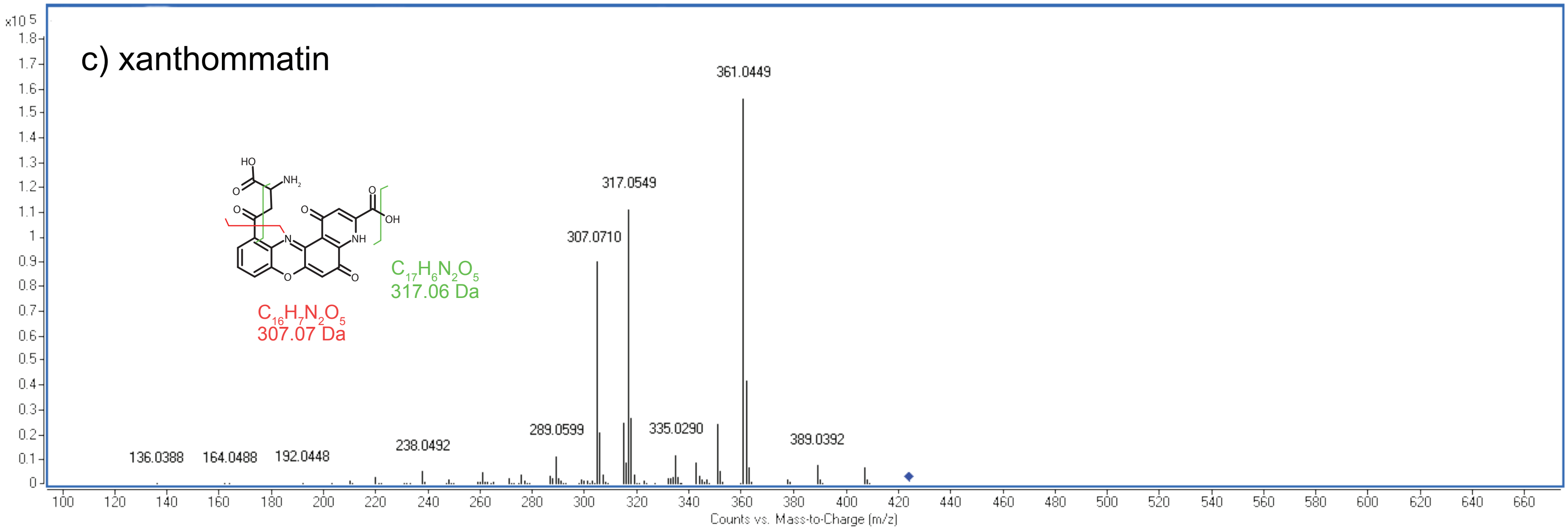

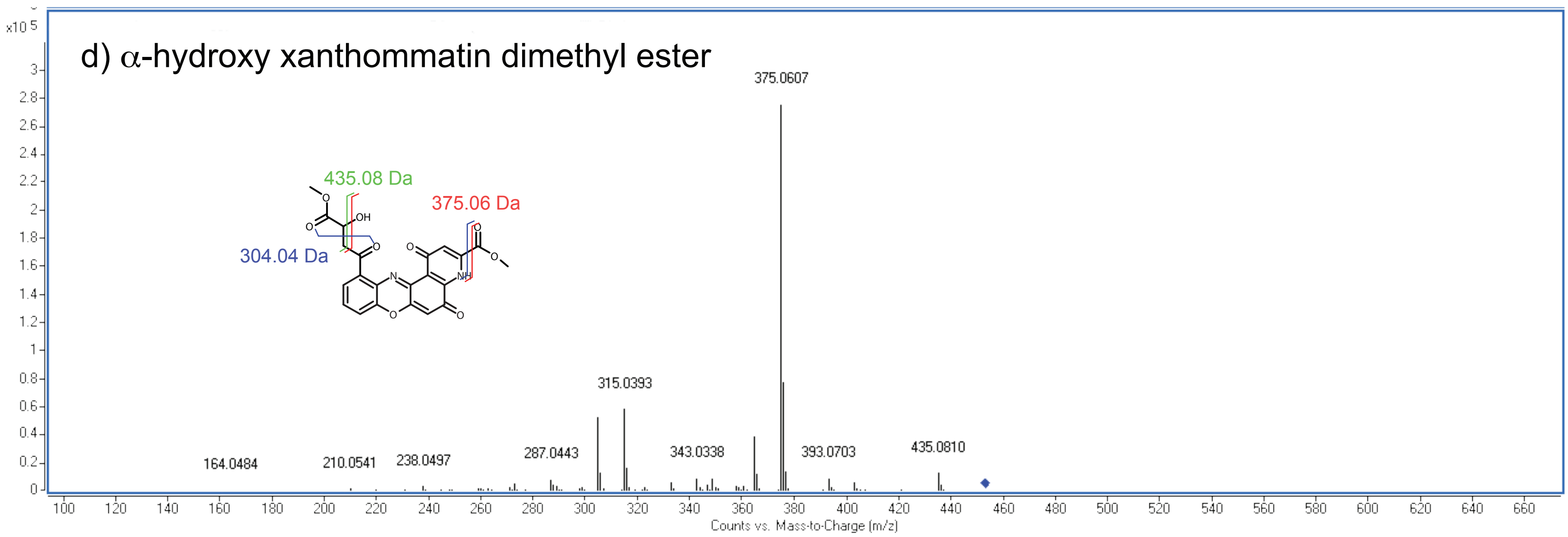
x10 5
d) α-hydroxy xanthommatin dimethyl ester
375.0607
435.08 Da
375.06 Da
304.04 Da
315.0393
164.0484
210.0541
238.0497
287.0443
343.0338
393.0703
435.0810
Counts vs. Mass-to-Charge (m/z)

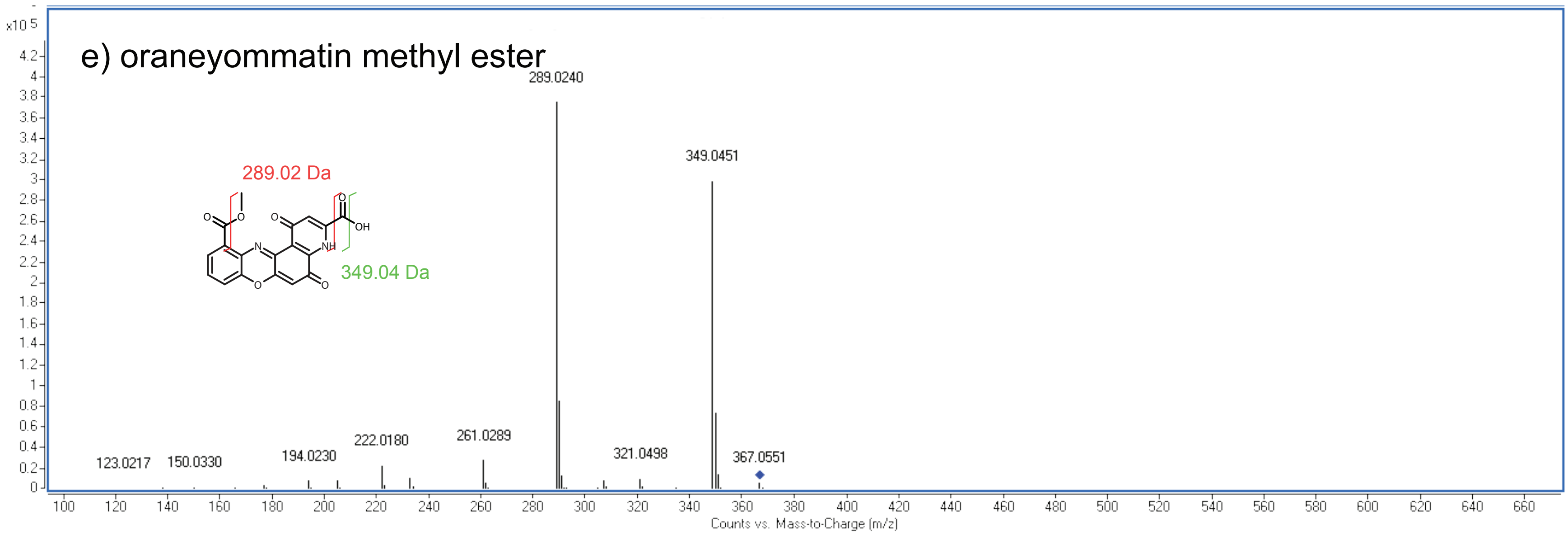
x10 5
e) oraneyommatin methyl ester
289.0240
349.0451
289.02 Da
349.04 Da
123.0217
150.0330
194.0230
222.0180
261.0289
321.0498
367.0551
Counts vs. Mass-to-Charge (m/z)

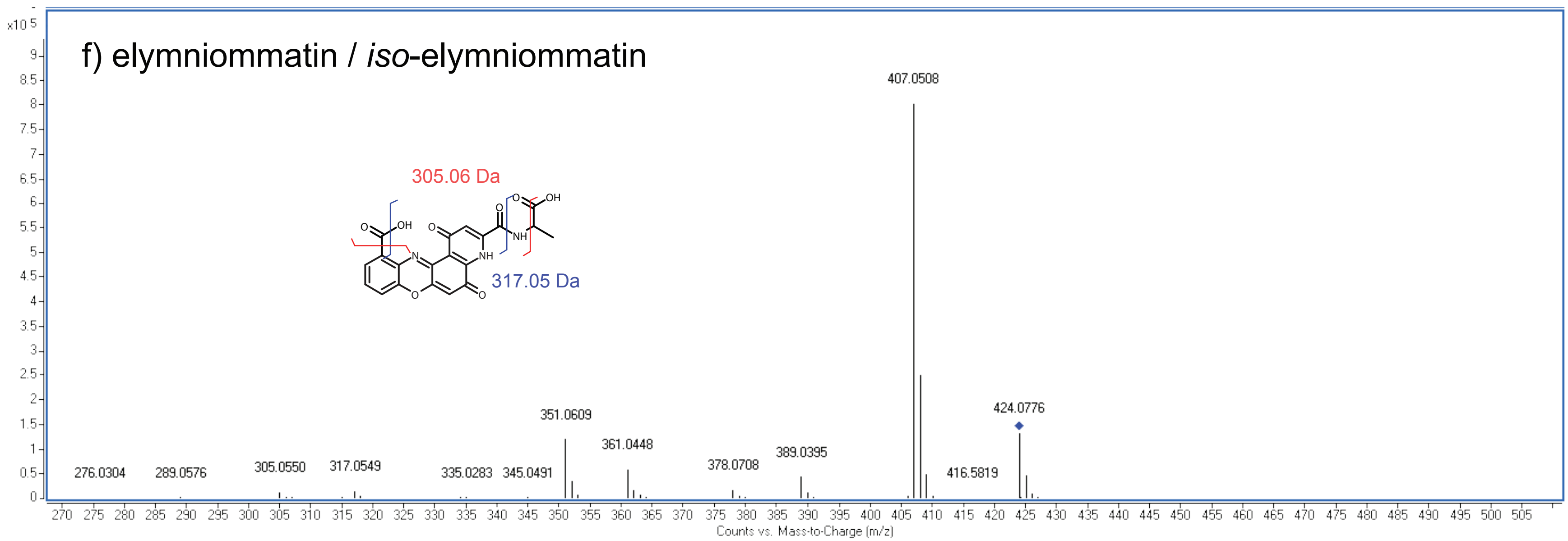
f) elymniommatin / iso-elymniommatin
x10 5
305.06 Da
317.05 Da
407.0508
424.0776
351.0609
361.0448
378.0708
389.0395
416.5819
276.0304
289.0576
305.0550
317.0549
335.0283
345.0491
Counts vs. Mass-to-Charge (m/z)

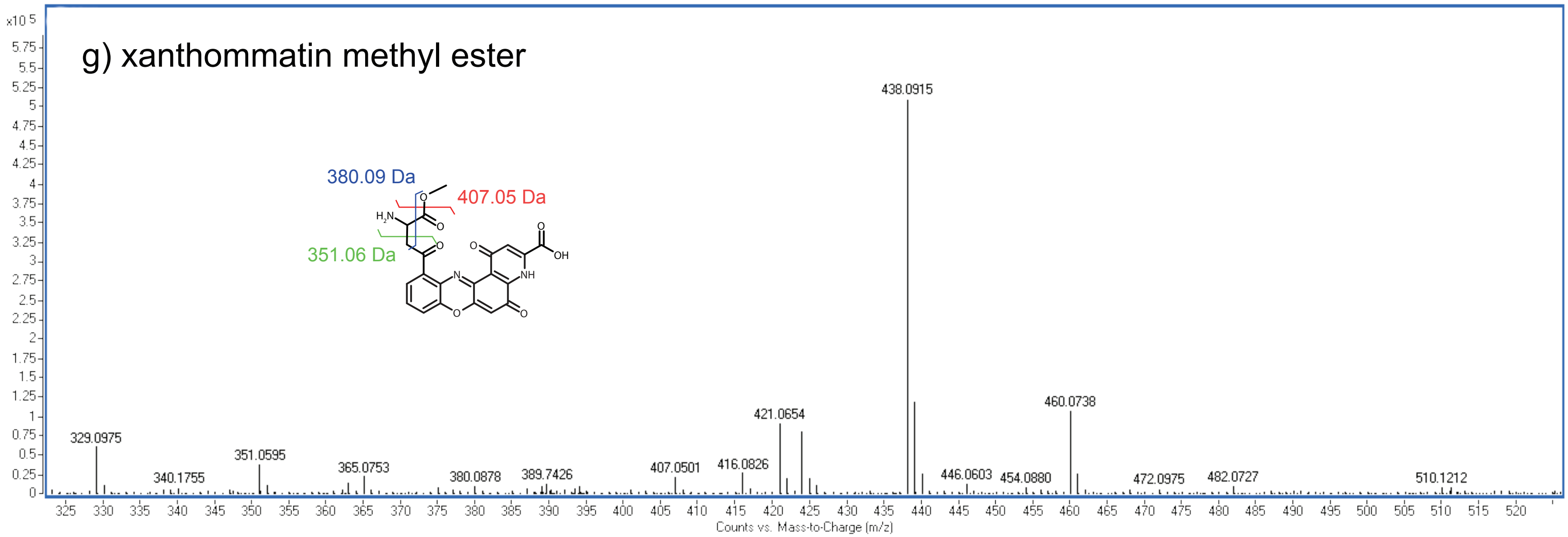
g) xanthommatin methyl ester
x10 5
380.09 Da
407.05 Da
351.06 Da
438.0915
460.0738
421.0654
329.0975
340.1755
351.0595
365.0753
380.0878
389.7426
407.0501
416.0826
446.0603
454.0880
472.0975
482.0727
510.1212
Counts vs. Mass-to-Charge (m/z)

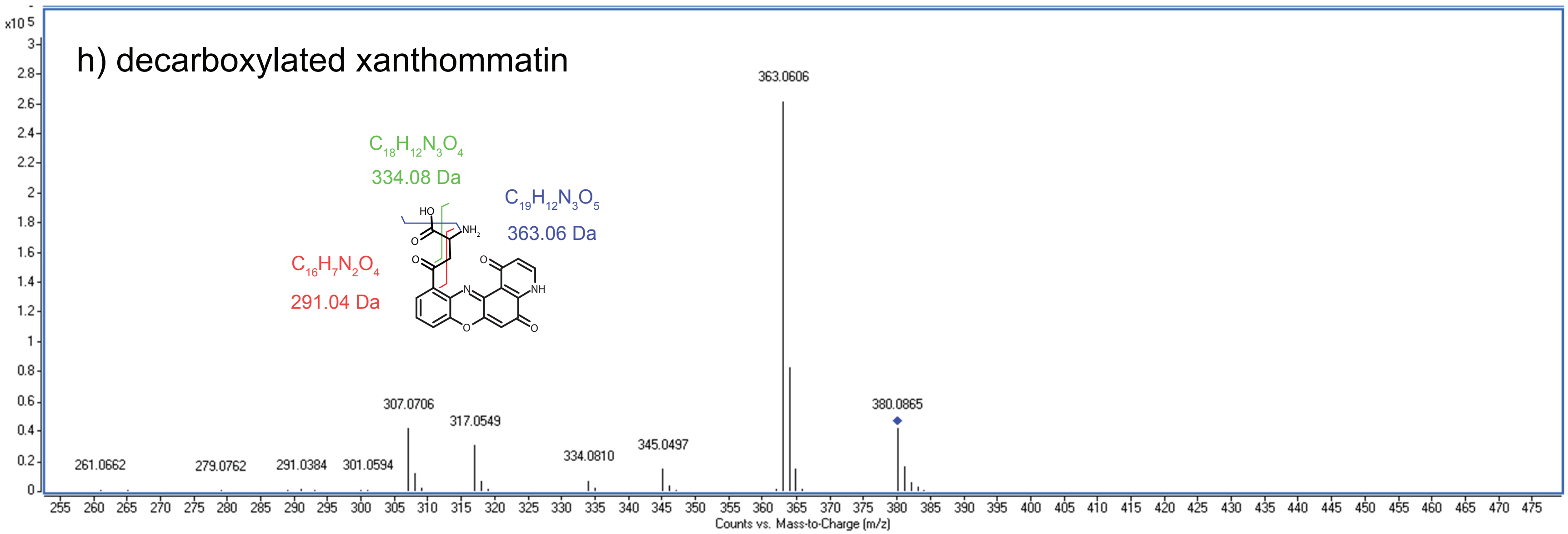

h) decarboxylated xanthommatin
$C_{18}H_{12}N_3O_4$
334.08 Da
$C_{19}H_{12}N_3O_5$
363.06 Da
$C_{16}H_7N_2O_4$
291.04 Da
363.0606
307.0706
317.0549
380.0865
334.0810
345.0497
261.0662
279.0762
291.0384
301.0594
x10 5
Counts vs. Mass-to-Charge (m/z)


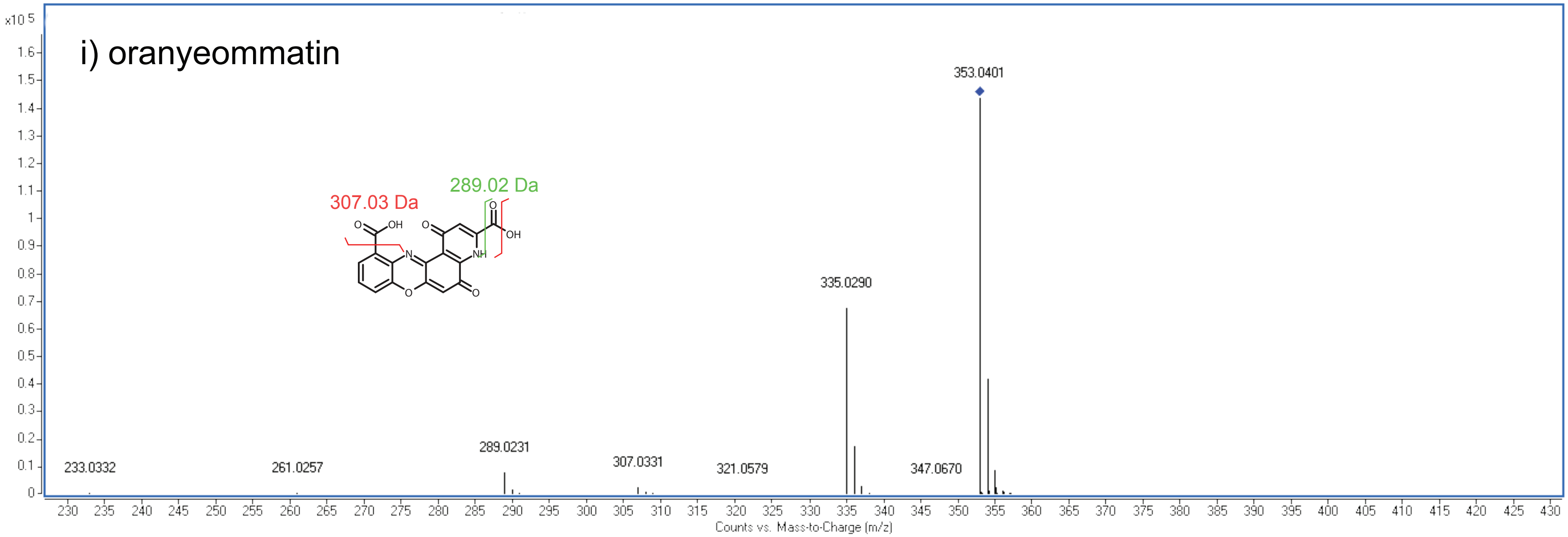

i) oranyeommatin
307.03 Da
289.02 Da
353.0401
335.0290
289.0231
307.0331
321.0579
347.0670
233.0332
261.0257
x10 5
Counts vs. Mass-to-Charge (m/z)

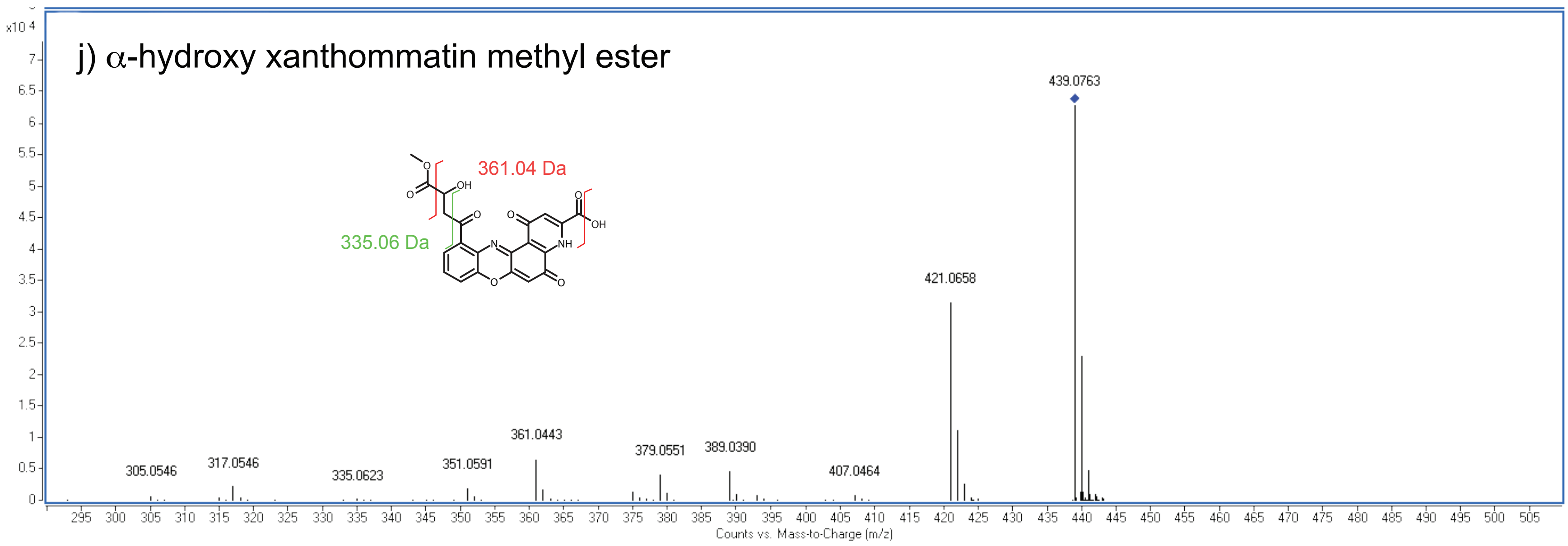

j) α-hydroxy xanthommatin methyl ester
x10 4
361.04 Da
335.06 Da
439.0763
421.0658
305.0546
317.0546
335.0623
351.0591
361.0443
379.0551
389.0390
407.0464
Counts vs. Mass-to-Charge (m/z)
